# Patterns of atopic eczema disease activity from birth through mid-adulthood in two British birth cohorts

**DOI:** 10.1001/jamadermatol.2021.2489

**Published:** 2021-10-01

**Authors:** Katrina Abuabara, Morgan Ye, David J. Margolis, Charles E McCulloch, Amy R Mulick, Richard J. Silverwood, Alice Sullivan, Hywel C. Williams, Sinéad M. Langan

**Affiliations:** 1 Program for Clinical Research, Department of Dermatology, University of California, San Francisco School of Medicine (UCSF), San Francisco, CA, USA; 2 Department of Dermatology and Center for Epidemiology and Biostatistics, University of Pennsylvania Perelman School of Medicine, Philadelphia, PA, USA; 3 Division of Biostatistics, University of California, San Francisco School of Medicine (UCSF), San Francisco, CA, USA; 4 Faculty of Epidemiology and Population Health, London School of Hygiene and Tropical Medicine, Keppel Street, London, WC1E 7HT, UK; 5 Centre for Longitudinal Studies, UCL Social Research Institute, University College London, UK; 6 Centre of Evidence Based Dermatology, Faculty of Medicine & Health Sciences, University of Nottingham

## Abstract

**Importance:**

Atopic eczema is characterized by a heterogenous waxing and waning course, with variable age of onset and persistence of symptoms. Distinct patterns of disease activity such as early-onset/resolving and persistent disease have been identified throughout childhood; little is known about patterns into adulthood.

**Objectives:**

To identify subtypes of atopic eczema based on patterns of disease activity through mid-adulthood, to examine whether early life risk factors and participant characteristics predict these subtypes, and to determine whether subtypes are associated with other atopic diseases and general health in mid-adulthood.

**Design:**

Two population-based birth cohort studies.

**Setting:**

United Kingdom, 1958-2016.

**Participants:**

Members of the 1958 National Childhood Development Study (NCDS) and the 1970 British Cohort Study (BCS70).

**Main outcomes and measures:**

Subtypes of atopic eczema patterns were identified based on self-reported atopic eczema period prevalence at multiple occasions. These subtypes were the outcome in models of early life characteristics and an exposure variable in models of mid-life health.

**Results:**

Latent class analysis identified four subtypes with distinct patterns of disease activity among 15,939 individuals from the NCDS and 14,966 individuals from the BCS70: ‘rare/no’ atopic eczema (88-91%), ‘decreasing’ (4%), ‘increasing’ (2-6%), and persistently ‘high’ (2-3%) probability of reporting prevalent atopic eczema with age. Early life factors, including sex at birth, social class, region of residence, tobacco smoke exposure, and breastfeeding, predicted differences between the three atopic eczema subtypes and the infrequent/no atopic eczema group, but only female sex differentiated the high and decreasing probability subtypes. Individuals in the high subtype were most likely to experience asthma and rhinitis, and those in the increasing subtype were at higher risk of poor self-reported general (OR 1.29, 95%CI 1.09 to 1.53) and mental (OR 1.45, 95%CI 1.23 to 1.72) health in mid-life.

**Conclusions and Relevance:**

Extending the window of observation beyond childhood, we observed clear subtypes of atopic eczema based on patterns of disease activity. A newly identified subtype with increasing probability of activity in adulthood warrants additional attention given observed associations with poor self-reported health in mid-life.

## Introduction

Atopic eczema (also known as atopic dermatitis or eczema)^1^ affects up to 20% of children in industrialized settings,^2-5^ and is characterized by a heterogenous waxing and waning course, with variable age of onset and persistence of symptoms. Prior studies have identified differences in patterns of disease activity throughout childhood. In particular, subtypes with resolving disease in childhood have been identified, and early onset during the first two years of life has been associated with genetic variants and development of asthma and allergies.^6-10^

Newer research has shown that atopic eczema is also common among adults, affecting 7-10% of the UK and US populations.^11-13^ There is a lack of studies that prospectively examine the course of atopic eczema beyond adolescence/early adulthood, and a more comprehensive understanding of disease activity across the lifespan is needed.^14,15^ Data on long-term disease course may offer insight into mechanisms for disease onset and persistence, are important when counselling patients, and would establish baseline trajectories for future studies of whether new treatments can modify disease course and development of comorbidities.

Using two British population-based longitudinal birth cohorts with over 40 years of follow-up, we previously found that the majority of adults with atopic eczema had symptom onset after childhood, and that there were important differences in risk factor profiles between those with childhood-onset and adult-onset disease.^16^ However, this study may not fully represent the real-world heterogeneity in disease course.

Herein our objectives were to identify subtypes of atopic eczema based on patterns of disease activity from birth through mid-adulthood, to examine whether early life risk factors and participant characteristics predict these subtypes, and to determine whether the subtypes are associated with other atopic diseases and general health in mid-adulthood.

## Methods

### Study population

We used data from the 1958 National Child Development Study (NCDS) and 1970 British Cohort Study (BCS70), which are ongoing, multidisciplinary studies that include 17,415 and 17,196 individuals born in Great Britain during one week in March 1958 and March 1970, respectively.^17,18^ There have been 9 subsequent waves of follow up in each cohort at approximately 5-10 year intervals. In the NCDS, we excluded the waves at ages 33, 46, and 55 because atopic eczema data were not collected, and we excluded individuals without any data on the presence/absence of symptoms of atopic eczema (N=1,476 for NCDS; N=2,230 for BCS70). Additional information on response patterns in both cohorts has been reported elsewhere.^19,20^

### Identification of atopic eczema subtypes based on disease activity over time

We used latent class analysis (LCA) to identify subtypes of atopic eczema activity patterns based on parental or self-reported atopic eczema period prevalence from standardized questions \ at 6 (NCDS) to 9 (BCS70) waves of follow up (eTable 1). This measure was previously shown to coincide with standardized clinical examinations among children in the NCDS,^21,22^ and a similar question demonstrated high sensitivity and specificity for physician-diagnosed atopic eczema in US populations.^23^

We ran generalized linear latent and mixed models with increasing numbers of latent classes. To select the number of classes, we examined model fit statistics, the estimated class sizes, and clinical interpretation.^24^ Models with lower adjusted Bayesian Information Criteria (BIC) values and higher entropy values that produced subtypes including ≥2% of the population were preferred.

We estimated the posterior latent class (subtype) probabilities and examined their distributions. Probabilities close to 1 suggest a trajectory is well-classified and distinct from the other subtype trajectories. Individuals were assigned to the class for which they had the highest posterior probability. We visually evaluated the consistency of individual patterns of disease activity within each class using heatmaps with a dendrogram,

### Identification of early- and mid-life factors associated with atopic eczema subtypes

We examined associations between early life factors and the newly identified atopic eczema subtypes, which were considered categorical outcomes in multinomial logistic regression models. Early-life factors were selected *a priori* based on literature showing associations with childhood atopic eczema and adult-onset eczema in the British birth cohorts.^16,21,25,26^ Adjusted models included sex, ethnic group, history of any breastfeeding, region of residence in childhood, childhood smoke exposure (either parent reporting current smoking at age 5 in the BCS70, and age 16 in the NCDS), household size (<=3 persons vs. 4+ persons), maternal in utero smoke exposure, birth weight, and the Registrar General’s designation of social class in childhood (a standard measure based on the father’s highest occupational status reported on any survey at ages 0-10/11). Data on parental history of asthma and hay fever were only available in the BCS70 at age 5 years.

Next, we examined whether atopic eczema subtype (now as a predictor variable) was associated with binary outcomes of self-reported asthma, hay fever, general health, and mental health in separate multivariable logistic regression models adjusted for sex, ethnicity, social class, and cohort. Asthma and rhinitis were based on self-report of the condition at age 46 (BCS70) or age 50 (NCDS, eTable1). General health was assessed using the 36-Item Short Form Health Survey (SF-36) at age 50 (NCDS) and age 46 (BCS70).^27^ We used a derived score based on five questions in the general health domain that ranged from 1 to 100, with higher scores indicating a more positive self-assessment of health, as the outcome in a linear regression model.^28^ To compare with other analyses, we also dichotomized responses from a single question from the SF-36 about general health (“Excellent/very good/good” vs “fair/poor”). Mental health was assessed using the Malaise Inventory scale at age 42. Based on previous literature, a cut-off of >=7 (of 24 items) and >=4 (of nine items) was used to define high risk of psychiatric morbidity for NCDS and BCS70, respectively.^29^

We performed all regressions separately in each cohort, and after evaluating for consistency, we conducted a meta-analysis of individual participant data, assuming fixed effects across studies and accounting for the clustering of participants within cohorts.

### Sensitivity analyses

Because the timing of questions about atopic eczema period prevalence varied slightly across cohorts (eTable 1), we performed a sensitivity analysis repeating the cohort specific LCAs using only five time points that were most consistent across both cohorts.

### Missing data

The LCA method accommodates missing outcome (atopic eczema) data with full information maximum likelihood estimation, therefore we used all available atopic eczema data in both cohorts and assessed individual patterns of missingness using heat maps. We performed multiple imputation in each cohort separately with iterative chained equations to impute missing early-life data (see supplemental methods for details). The imputed results were compared to the complete case analysis. We did not impute data on atopic comorbidities or general/mental health in mid-life because most of the missing data were due to attrition.

This study was exempt from IRB approval because no identifiable information was accessed by the study team. Statistical analyses were conducted using Stata, version 16 (StataCorp, College Station TX).

## Results

The study sample included 15,939 individuals from the NCDS and 14,966 from the BCS70. Atopic eczema was reported at ≥1 time point(s) by 16.7% and 24.4% of participants in the NCDS and BCS70, respectively. Demographic and early-life characteristics for both cohorts are presented in Table 1.

### Atopic eczema subtypes based on disease activity over time

Model fit statistics demonstrated that 5-class models performed slightly better than 4-class models, however, the additional class included only 1% of the sample in each cohort, resulted in lower entropy scores, and did not improve the clinical interpretation (eFigure 1 and eTable 2). Therefore, we present results for the four-class model. In this model, participants had a high probability of being assigned to their most likely subtype and a low probability of being in the other subtypes (eTable 3), and individual disease trajectories followed a similar overall pattern despite individual heterogeneity (eFigure 2).

**Figure 1:**
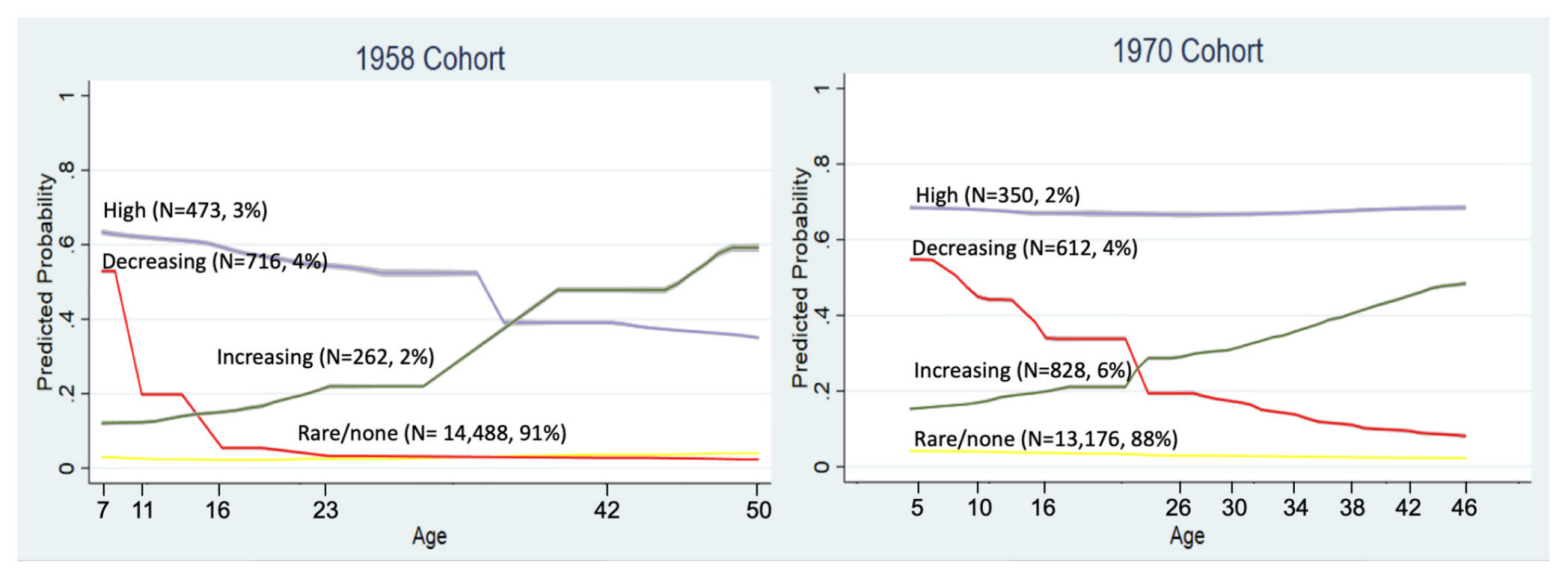
Estimated probabilities of atopic eczema symptoms at each age for each subtype in 4-class models from the NCDS and BCS70 cohorts Predicted probabilities at each age generated from generalized linear and latent mixed models

We labeled the subtypes in each cohort by the probability of atopic eczema with age: ‘rare/no atopic eczema’, ‘decreasing’, ‘high’, and ‘increasing’ (Figure 1). The decreasing and high subtypes were similar in size in both cohorts, but the increasing subtype was larger in the BCS70 as compared to the NCDS (6% vs 2%).

A sensitivity analysis using only atopic eczema data from the five time points most similar between cohorts did not achieve as good of a model fit or separation between the classes in mid-adulthood as analyses utilizing all available data (eTable 2).

### Early life factors as predictors of atopic eczema subtypes

We examined associations between atopic eczema subtypes and early life factors using rare/no atopic eczema as a reference group. We found that sex, region of residence, social class, in utero smoke exposure, and breastfeeding were associated with differences in disease trajectory subtype (Table 2). Female sex was associated with higher odds of the high and increasing subtypes and a lower odds of the decreasing subtype as compared to rare/no atopic eczema group. Those who resided in Northern England or Scotland during childhood experienced lower odds of all subtypes of atopic eczema than those who resided in Southern England. Those from higher childhood social class and who were breastfed were more likely to be in the high and decreasing subtypes. Parental history of atopic disease in the BCS70 was a significant predictor of all atopic eczema subtypes as compared to the rare/no atopic eczema group (eTable 4).

Using the same regression models but changing the comparison group to examine differences between atopic eczema subgroups, we found that only female sex was associated with an increased risk of the high subtype as compared to the decreasing subtype (Table 3). Residence in Central England as compared to Southern England was also associated with an increased risk of the high subtype, but the full geography variable did not meet statistical significance as an independent predictor (p=0.100). Female sex and lower childhood social class were associated with increasing as compared to decreasing subtypes.

### Associations between disease trajectory subtypes with other atopic disease, general health, and mental health in mid-life

Those classified into the highly probable atopic eczema subtype consistently had the highest prevalence of asthma/wheeze and rhinitis, and those classified into the no/rare atopic eczema subtype had the lowest rates (eTable 5). Compared to the no/rare atopic eczema subtype, all other subtypes had an increased odds of asthma and rhinitis in mid-life (Table 4). The increasing and high subtypes were both associated with worse self-reported general health, and only the increasing subtype was associated with poor mental health (Table 4).

### Sensitivity and missing data analyses

Primary results described above use the combined and imputed cohort. Additional analyses conducted separately in each cohort (eTable 4) and a complete case analysis (eTable 6) yielded similar results.

## Discussion

Leveraging two large population-based birth cohorts with repeated assessments of individual atopic eczema disease activity, we identified four distinct disease trajectories based on probability of reporting atopic eczema with age: a majority of people who report no atopic eczema or who report it rarely, decreasing probability, high probability, and increasing probability of reporting atopic eczema with age. Previously identified early life risk factors including sex, region of residence, parental social class, in utero smoke exposure and breastfeeding were associated with the atopic eczema subtypes as compared to those with rare or no atopic eczema, but only female sex helped to differentiate the high from decreasing subtype. A newly identified subtype with increasing probability of atopic eczema with age was most likely to have poor self-reported general and mental health in mid-life.

### Comparison to existing literature

There is a lack of clarity regarding the terminology around atopic eczema/atopic dermatitis/eczema. We chose to use the term ‘atopic eczema’ because ‘atopic dermatitis’ is less commonly used in the UK, and using the term ‘eczema’ alone is not recommended.^30^ The term ‘atopic’ does not necessarily indicate the presence of atopy; in many population-based cohorts, most patients do not demonstrate IgE-specific antibodies to common environmental allergens.^31^

We previously found differences between cohort participants who reported childhood-onset versus adulthood-onset atopic eczema.^16^ Our current analysis represents a substantial additional contribution because we used a hypothesis-free approach to identify distinct subtypes based on data from all ages and were able to compare predictors and outcomes between those subtypes. These subtypes describe age-associated probabilities, not individual disease trajectories. Thus, we chose probabilistic labels such as ‘increasing’ rather than ‘late-onset,’ since an individual in the increasing probability subtype may not necessarily have late-onset disease: 21% of individuals in NCDS and 27% in BCS70 classified into this subtype experienced atopic eczema symptoms at some point in childhood (prevalence of atopic eczema by subtype and age shown in eTable 5).

While other studies have examined atopic eczema subtypes based on disease activity across childhood;^6-10^ ours is unique in its duration of follow-up into mid-life which allowed for identification of a new subtype with an increasing probability of atopic eczema over time. This subtype was associated with poor general and mental health in mid-life. An Australian study that examined asthma trajectories over many decades similarly found that late-onset asthma was most strongly associated with general health.^32^ Other studies show that the immune profile of atopic eczema varies by age, but haven’t yet examined disease course.^33,34^ These results indicate that disease duration and activity may be important in predicting outcomes and should be included in studies of atopic eczema comorbidities.

Geographic and temporal differences suggest that environmental factors may influence disease course. Geographic location was associated with all the atopic eczema subtypes in our study. A prior study using NCDS data found that regional differences in both examined and reported eczema were maintained when data were analyzed at the county level.^22^ There were also temporal differences: atopic eczema was more common in the BCS70, which is consistent with secular trends documented in other data sources,^35,36^ and the increasing subtype was larger in the BCS70 (6% vs 2% in the NCDS). This may indicate it is becoming more common, though additional studies are needed to confirm this finding. Environmental factors including temperature and humidity, water hardness, pollution levels, and the environmental microbiome have been linked to AD and may vary geographically and temporally.^22,37-39^ Future research should examine the role of environmental factors on patterns and persistence of atopic eczema over time.

Breastfeeding was associated with a higher risk of all the atopic eczema subtypes in our study, and in utero smoke exposure was associated with a lower risk of the high subtype. In the literature, there have been mixed findings on these factors.^40,41^ Women with a history of atopy may be less likely to smoke and more likely to breastfeed, which could explain our results. Additionally, the timing and duration of smoking and breastfeeding are likely to be important, as well as potential confounding by social factors we were unable to control for in our study. Given the constraints of historical data analysis, we were unable to further examine these issues. The health risks of smoking and benefits of breastfeeding are both well-established,^42,43^ and our results should not be interpreted as implying otherwise.

### Limitations

While our study relied on two large, generalizable cohorts with five decades of follow-up, it was also limited by missing data and lack of clinical detail. We performed multiple imputation to address missing early life data and did not identify patterns indicative of bias due to attrition. The reliance on self-report of atopic eczema could result in misclassification bias. Self-report has been shown to reasonably approximate clinical assessment in childhood^21,22^ and physician diagnosis in adulthood,^23^ and is unlikely to be differential over time among individuals. Both cohorts had similar population-based designs, but the wording and timing of questions differed between cohorts (eTable1). A prior investigation did not find evidence of bias due to changes in study design.^44^ A methodological limitation is that allocating individuals to their most likely latent class then analyzing these classes as if they were observed may overstate precision in the analytical models. However, given the high level of entropy and average posterior class membership probabilities, this is unlikely to be a major limitation.

We could not examine atopic eczema severity or how treatment patterns affect disease course, which is an important area for future research. For example, among adults with mild asthma, early initiation of inhaled corticosteroid treatment may improve long-term outcomes. As more systemic treatments become available for atopic eczema,^45^ randomized trials to examine whether these treatments affect disease trajectory are of utmost importance. Our results help establish a baseline for long-term disease course.

### Conclusion

When extending the window of observation beyond childhood, clear subtypes of atopic eczema based on patterns of disease activity emerged. In particular, a newly identified subtype with increasing probability of activity in adulthood warrants additional attention given associations with poor self-reported physical and mental health in mid-life. Early life factors shown to be associated with childhood atopic eczema overall did not help differentiate between subtypes raising the possibility that disease trajectory is modifiable and may be influenced by environmental factors throughout life.

## Supplementary Material

Supplementary

## Figures and Tables

**Table 1:** Patient characteristics by cohort

	NCDS^a^			BCS70^b^		
	Total N=15,939^c^	No atopic eczema N=13,278 (83.3%)	Atopic eczema N=2,661 (16.7%)	Total N=14,966^c^	No atopic eczema N=11,310 (75.6%)	Atopic eczema N=3,656 (24.4%)
Asthma						
No	12633 (79.3%)	10809 (81.4%)	1824 (68.5%)	11996 (80.2%)	9359 (82.7%)	2637 (72.1%)
Any	3238 (20.3%)	2402 (18.1%)	836 (31.4%)	2300 (15.4%)	1355 (12.0%)	945 (25.8%)
Missing	68 (0.4%)	67 (0.5%)	1 (0.0%)	670 (4.5%)	596 (5.3%)	74 (2.0%)
Rhinitis						
No	11718 (73.5%)	10140 (76.4%)	1578 (59.3%)	9624 (64.3%)	7836 (69.3%)	1788 (48.9%)
Any	4149 (26.0%)	3066 (23.1%)	1083 (40.7%)	5329 (35.6%)	3467 (30.7%)	1862 (50.9%)
Missing	72 (0.5%)	72 (0.5%)	0 (0.0%)	13 (0.1%)	7 (0.1%)	6 (0.2%)
Sex						
Male	8200 (51.4%)	6996 (52.7%)	1204 (45.2%)	7716 (51.6%)	6110 (54.0%)	1606 (43.9%)
Female	7739 (48.6%)	6282 (47.3%)	1457 (54.8%)	7250 (48.4%)	5200 (46.0%)	2050 (56.1%)
Ethnicity						
White	12018 (75.4%)	9843 (74.1%)	2175 (81.7%)	11790 (78.8%)	8758 (77.4%)	3032 (82.9%)
Other	160 (1.0%)	132 (1.0%)	28 (1.1%)	510 (3.4%)	389 (3.4%)	121 (3.3%)
Missing	3761 (23.6%)	3303 (24.9%)	458 (17.2%)	2666 (17.8%)	2163 (19.1%)	503 (13.8%)
Region of residence in childhood						
Southern England	4871 (30.6%)	4014 (30.2%)	857 (32.2%)	4427 (29.6%)	3177 (28.1%)	1250 (34.2%)
Central England	4689 (29.4%)	3866 (29.1%)	823 (30.9%)	3454 (23.1%)	2555 (22.6%)	899 (24.6%)
Northern England	6379 (40.0%)	5398 (40.7%)	981 (36.9%)	4804 (32.1%)	3718 (32.9%)	1086 (29.7%)
Missing	0 (0.0)	0 (0.0%)	0 (0.0%)	2281 (15.3%)	1860 (16.4%)	421 (11.5%)
Social class in childhood^d^						
I/II	4489 (28.2%)	3632 (27.4%)	857 (32.2%)	5281 (35.3%)	3780 (33.4%)	1501 (41.1%)
IIIa/b	9626 (60.4%)	8069 (60.8%)	1557 (58.5%)	8410 (56.2%)	6504 (57.5%)	1906 (52.1%)
IV/V	1718 (10.8%)	1479 (11.1%)	239 (9.0%)	1238 (8.3%)	995 (8.8%)	243 (6.6%)
Missing	106 (0.7%)	98 (0.7%)	8 (0.3%)	37 (0.2%)	31 (0.3%)	6 (0.2%)
Household size						
<=3 persons	1211 (7.6)	996 (7.5%)	215 (8.1%)	1339 (8.9)	999 (8.8%)	340 (9.3%)
4+ persons	12357 (77.5)	10186 (76.7%)	2171 (81.6%)	11374 (76.0)	8471 (74.9%)	2903 (79.4%)
Missing	2371 (14.9)	2096 (15.8%)	275 (10.3%)	2253 (15.1)	1840 (16.3%)	413 (11.3%)
Smoking during pregnancy						
No	10482 (65.8%)	8690 (65.4%)	1792 (67.3%)	8050 (53.8%)	5963 (52.7%)	2087 (57.1%)
Any	5257 (33.0%)	4427 (33.3%)	830 (31.2%)	6847 (45.8%)	5293 (46.8%)	1554 (42.5%)
Missing	200 (1.3%)	161 (1.2%)	39 (1.5%)	69 (0.5%)	54 (0.5%)	15 (0.4%)
Childhood smoke exposure						
No	2993 (18.8%)	2424 (18.3%)	569 (21.4%)	4340 (29.0%)	3165 (28.0%)	1175 (32.1%)
Any	7953 (49.9%)	6564 (49.4%)	1389 (52.2%)	8337 (55.7%)	6272 (55.5%)	2065 (56.5%)
Missing	4993 (31.3%)	4290 (32.3%)	703 (26.4%)	2289 (15.3%)	1873 (16.6%)	416 (11.4%)
Parental history of atopy						
No	---	---	---	8638 (57.7%)	6687 (59.1%)	1951 (53.4%)
Any	---	---	---	2958 (19.8%)	1918 (17.0%)	1040 (28.4%)
Missing	---	---	---	3370 (22.5%)	2705 (23.9%)	665 (18.2%)
Birth weight (kg), mean (SD)	3.3 (0.5)	3.3 (0.5)	3.3 (0.5)	3.3 (0.5)	3.3 (0.5)	3.3 (0.5)
Missing	529 (3.3%)	445 (3.4%)	84 (3.2%)	14 (0.1%)	13 (0.1%)	1 (0.03%)
Breastfeeding						
No	4435 (27.8%)	3742 (28.2%)	693 (26.0%)	7957 (53.2%)	6075 (53.7%)	1882 (51.5%)
Any	9583 (60.1%)	7832 (59.0%)	1751 (65.8%)	4659 (31.1%)	3323 (29.4%)	1336 (36.5%)
Missing	1921 (12.1%)	1704 (12.8%)	217 (8.2%)	2350 (15.7%)	1912 (16.9%)	438 (12.0%)

**Notes:**

a National Childhood Development Survey

b 1970 British Cohort Study

c N is based on patients with at least one response to an eczema question, missing refers to number of individuals without data on each covariate.

d Registrar General’s social class: I Professional, II Managerial and technical; III Skilled; IV Partly-skilled; V Unskilled.

**Table 2:** Association between early life factors and subtypes as compared to rare/no AE subtype (results of 1958 and 1970 meta-analysis) (Total N across all classes = 30,905)

	High vs Rare/no AE	Decreasing vs Rare/no AE	Increasing vs Rare/no AE
	OR (95% CI)		
**Demographic / Early life factors**			
Sex			
Male	Reference	Reference	Reference
Female	**1.76 (1.53, 2.04)**	**0.89 (0.79, 0.99)**	**1.77 (1.56, 2.01)**
Ethnicity			
European, Caucasian	Reference	Reference	Reference
Other	0.89 (0.53, 1.48)	1.03 (0.72, 1.46)	0.86 (0.59, 1.25)
Region of early childhood residence^a^			
Southern England	Reference	Reference	Reference
Central England/Wales	1.06 (0.89, 1.27)	**0.85 (0.74, 0.97)**	0.93 (0.80, 1.09)
N. England/Scotland	**0.82 (0.69, 0.98)**	**0.69 (0.60, 0.79)**	**0.82 (0.70, 0.96)**
Highest social class in childhood^b,c^			
I/II	Reference	Reference	Reference
III	**0.69 (0.59, 0.80)**	**0.80 (0.71, 0.91)**	0.92 (0.80, 1.05)
IV/V	**0.50 (0.37, 0.68)**	**0.56 (0.44, 0.71)**	0.88 (0.69, 1.12)
Household size in early childhood			
<=3 persons	Reference	Reference	Reference
4+ persons	1.02 (0.79, 1.32)	0.95 (0.79, 1.15)	0.93 (0.75, 1.16)
In utero smoke exposure			
No	Reference	Reference	Reference
Any	**0.83 (0.70, 0.98)**	1.00 (0.87, 1.13)	0.91 (0.79, 1.05)
Childhood smoke exposure			
No	Reference	Reference	Reference
Any	0.94 (0.78, 1.12)	0.95 (0.82, 1.10)	0.91 (0.79, 1.06)
Birth weight			
Per kg increase	1.09 (0.95, 1.25)	1.01 (0.91, 1.13)	0.93 (0.83, 1.05)
Breastfeeding			
No	Reference	Reference	Reference
Any	**1.39 (1.18, 1.63)**	**1.20 (1.06, 1.36)**	**1.18 (1.03, 1.35)**

a Region of early childhood residence: overall p-values are 0.01 for high vs rare/no AE, <0.001 for decreasing vs rare/no AE, and 0.04 for increasing vs rare/no AE

b Registrar General’s social class: I Professional, II Managerial and technical; III Skilled; IV Partly-skilled; V Unskilled.

c Highest social class in childhood: overall p-values are <0.001 for high vs rare/no AE, <0.001 for decreasing vs rare/no AE, and 0.36 for increasing vs rare/no AE

**Table 3:** Association between early life factors and atopic eczema activity subtypes as compared to the early onset/decreasing subtype for the 1958 and 1970 cohorts meta-analysis (Total N across all classes = 30,905)

	High vs Decreasing AE	Increasing vs Decreasing AE
	OR (95% CI)	
Sex		
Male	Reference	Reference
Female	**1.99 (1.66, 2.38)**	**1.99 (1.69, 2.35)**
Ethnicity		
European, Caucasian	Reference	Reference
Other	0.86 (0.47, 1.58)	0.84 (0.51, 1.38)
Region of early childhood residence^a^		
Southern England	Reference	Reference
Central England/Wales	**1.26 (1.01, 1.56)**	1.10 (0.90, 1.36)
N. England/Scotland	1.18 (0.95, 1.47)	1.19 (0.97, 1.45)
Highest social class in childhood^b,c^		
I/II	Reference	Reference
III	0.86 (0.71, 1.03)	1.14 (0.95, 1.36)
IV/V	0.90 (0.61, 1.32)	**1.58 (1.13, 2.21)**
Household size in early childhood		
<=3 persons	Reference	Reference
4+ persons	1.08 (0.79, 1.47)	0.98 (0.75, 1.30)
In utero smoke exposure		
No	Reference	Reference
Any	0.83 (0.68, 1.03)	0.91 (0.76, 1.10)
Childhood smoke exposure		
No	Reference	Reference
Any	0.99 (0.79, 1.23)	0.96 (0.79, 1.18)
Birth weight		
Per kg increase	1.08 (0.90, 1.28)	0.92 (0.79, 1.08)
Breastfeeding		
No	Reference	Reference
Any	1.16 (0.95, 1.41)	0.98 (0.82, 1.17)
	High vs Decreasing AE	Increasing vs Decreasing AE
	OR (95% CI)	
Sex		
Male	Reference	Reference
Female	**1.99 (1.66, 2.38)**	**1.99 (1.69, 2.35)**
Ethnicity		
European, Caucasian	Reference	Reference
Other	0.86 (0.47, 1.58)	0.84 (0.51, 1.38)
Region of early childhood residence^a^		
Southern England	Reference	Reference
Central England/Wales	**1.26 (1.01, 1.56)**	1.10 (0.90, 1.36)
N. England/Scotland	1.18 (0.95, 1.47)	1.19 (0.97, 1.45)
Highest social class in childhood^b,c^		
I/II	Reference	Reference
III	0.86 (0.71, 1.03)	1.14 (0.95, 1.36)
IV/V	0.90 (0.61, 1.32)	**1.58 (1.13, 2.21)**
Household size in early childhood		
<=3 persons	Reference	Reference
4+ persons	1.08 (0.79, 1.47)	0.98 (0.75, 1.30)
In utero smoke exposure		
No	Reference	Reference
Any	0.83 (0.68, 1.03)	0.91 (0.76, 1.10)
Childhood smoke exposure		
No	Reference	Reference
Any	0.99 (0.79, 1.23)	0.96 (0.79, 1.18)
Birth weight		
Per kg increase	1.08 (0.90, 1.28)	0.92 (0.79, 1.08)
Breastfeeding		
No	Reference	Reference
Any	1.16 (0.95, 1.41)	0.98 (0.82, 1.17)

a Region of early childhood residence: overall p-values are 0.10 for high vs decreasing AE and 0.24 for increasing vs decreasing AE

b Registrar General’s social class: I Professional, II Managerial and technical; III Skilled; IV Partly-skilled; V Unskilled.

c Highest social class in childhood: overall p-values are 0.27 for high vs decreasing AE and 0.02 for increasing vs decreasing AE

**Table 4:** Associations between atopic eczema subtypes and other atopic disease, general health, and mental health in mid-life for the 1958 and 1970 meta-analysis

	Asthma N=17,200	Rhinitis N=17,200	Poor mental health at age 42 N=17,978	Poor general health at age 46/50 N=18,733	SF-36 General Health at age 46/50 N=15,810
	OR (95% CI)				Mean difference (95% CI)
Rare/no AE	Reference	Reference	Reference	Reference	Reference
High AE	**3.45 (2.82, 4.21)**	**2.70 (2.24, 3.26)**	1.06 (0.85, 1.32)	**1.26 (1.03, 1.55)**	-1.24 (-3.21, 0.73)
Decreasing AE	**1.70 (1.38, 2.10)**	**1.42 (1.18, 1.70)**	0.89 (0.73, 1.08)	0.88 (0.72, 1.07)	0.03 (-1.67, 1.73)
Increasing AE	**2.11 (1.75, 2.53)**	**1.92 (1.64, 2.25)**	**1.45 (1.23, 1.72)**	**1.29 (1.09, 1.53)**	**-4.52 (-6.12, -2.92)**

Decreasing AE	Reference	Reference	Reference	Reference	Reference
High AE	**2.02 (1.53, 2.68)**	**1.91 (1.48, 2.46)**	1.20 (0.90, 1.61)	**1.44 (1.09, 1.90)**	-1.27 (-3.82, 1.28)
Increasing AE	1.24 (0.95, 1.62)	**1.36 (1.07, 1.72)**	**1.64 (1.27, 2.11)**	**1.47 (1.15, 1.89)**	**-4.55 (-6.83, -2.27)**

Increasing AE	Reference	Reference	Reference	Reference	Reference
Decreasing AE	0.81 (0.62, 1.06)	**0.74 (0.58, 0.93)**	**0.61 (0.47, 0.79)**	**0.68 (0.53, 0.87)**	**4.55 (2.27, 6.83)**
High AE	**1.63 (1.26, 2.12)**	**1.41 (1.11, 1.79)**	**0.73 (0.56, 0.96)**	0.98 (0.75, 1.27)	**3.28 (0.79, 5.77)**

a Each column represents a separate logistic or linear regression model predicting the odds of each health outcome. The rows shows different reference groups for the same regression model.

## Key points

- **Question:** Are there patterns of atopic eczema activity into adulthood?
- **Findings:** Among over 30,905 participants followed from birth into mid-life four patterns of activity were identified: high probability, decreasing, increasing, and low probability of atopic eczema across ages. Early life factors did not differentiate the active subtypes, and the subtype with increasing probability of activity had the highest risk of poor self-reported health in mid-life.
- **Meaning:** The probability of atopic eczema remains high into adulthood for a subgroup of patients, and a newly identified subtype with increasing probability of activity in adulthood warrants additional attention.
