# Supplementary material for: Patterns of atopic eczema disease activity from birth through mid-adulthood in two British birth cohorts
Source: JAMA Dermatol. Author manuscript; Available in PMC 2021 Oct 22. (PMC8411362; doi:10.1001/jamadermatol.2021.2489)
Supplement: Supplementary [file EMS133994-supplement-Supplementary.docx]

**Supplement to: Patterns of atopic eczema disease activity from birth through mid-adulthood in two British birth cohorts**

Katrina Abuabara, MD, MA, MSCE^1^

Morgan Ye, MPH^1^

David J. Margolis, MD, PhD^2^

Charles E McCulloch, PhD^3^

Amy R Mulick MSc^4^

Richard J. Silverwood, PhD^5^

Alice Sullivan, PhD^5^

Hywel C. Williams, FRCP, DSc^6^

Sinéad M. Langan, FRCP, PhD^4^

1. Program for Clinical Research, Department of Dermatology, University of California, San Francisco School of Medicine (UCSF), San Francisco, CA, USA
2. Department of Dermatology and Center for Epidemiology and Biostatistics, University of Pennsylvania Perelman School of Medicine, Philadelphia, PA, USA
3. Division of Biostatistics, University of California, San Francisco School of Medicine (UCSF), San Francisco, CA, USA
4. Faculty of Epidemiology and Population Health, London School of Hygiene and Tropical Medicine, Keppel Street, London, WC1E 7HT, UK
5. Centre for Longitudinal Studies, UCL Social Research Institute, University College London, UK
6. Centre of Evidence Based Dermatology, Faculty of Medicine & Health Sciences, University of Nottingham

**Contents**

- **eMethods**
- **eFigure 1:** Subtype interpretations by model
- **eFigure 2** Heat maps of individual participant disease patterns across ages, organized by subgroup from the latent class analysis
- **eTable 1.** Questions about atopic eczema, asthma, and rhinitis by year and by cohort
- **eTable 2.** Latent class fit statistics
- **eTable 3:** Average classification probabilities by most likely disease trajectory subtype for the four-class solution
- **eTable 4:** Proportions of individuals with atopic eczema, asthma, and rhinitis within each disease trajectory subtype with by cohort
- **eTable 5.** Association between early life factors and atopic eczema activity subtypes by cohort
- **eTable 6.** Complete case analysis: Association between early life factors and atopic eczema activity subtypes from for 1958 and 1970 meta-analysis

**eMethods**

For the imputation analysis, thirty imputed datasets were generated. All of the eczema variables used to generate the latent classes and all early life factors were used to impute the missing variables. Auxilliary variables were also used to impute missing variables. For 1958, we included asthma, hayfever/rhinitis, contact dermatitis, adulthood social class, smoking during adulthood, mother’s age at birth, father’s age at birth, maternal education at birth, number of people living per room at birth, number of people mother cooked for when pregnant, antenatal visits, and parity. For 1970, we included asthma, hayfever/rhinitis, parent atopy, contact dermatitis, adulthood social class, smoking during adulthood, marital status at birth, mother’s age at completion of education at birth, premarital conception, method of labor, and lactation.

## **eFigure 1: Subtype interpretations by model**

Each column contains a description of the phenotypes resulting from latent class analyses (LCAs) with the indicated number of classes. Arrows indicate when >8% of individuals within a phenotype move to a new phenotype when an additional class is allowed into the LCA.

1958 Cohort


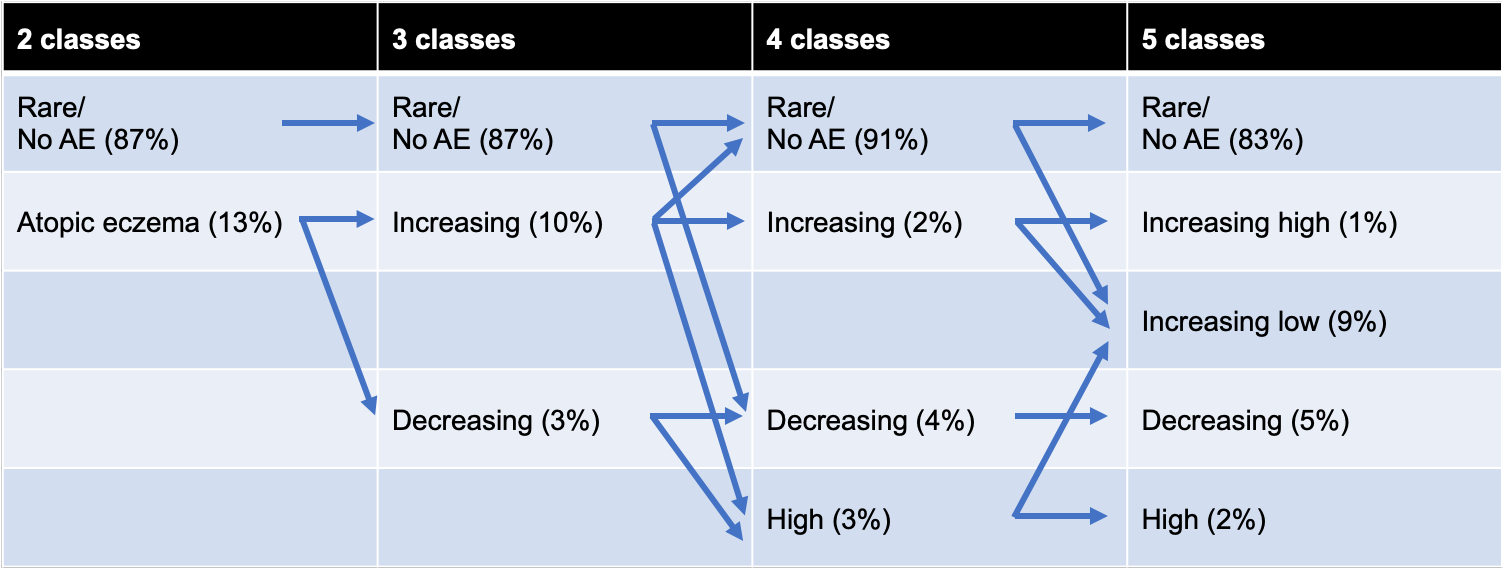


1970 Cohort


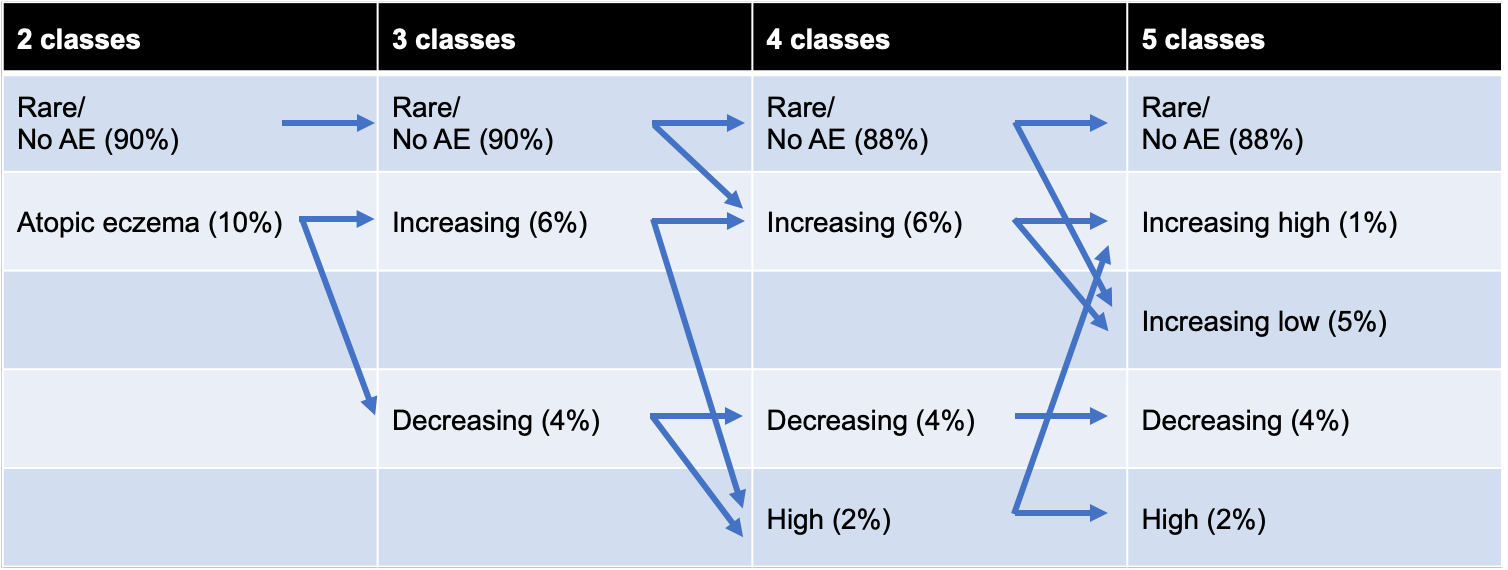


**eFigure 2. Heat maps of individual participant disease patterns across ages, organized by subgroup from the latent class analysis**

**1958 Cohort**

**
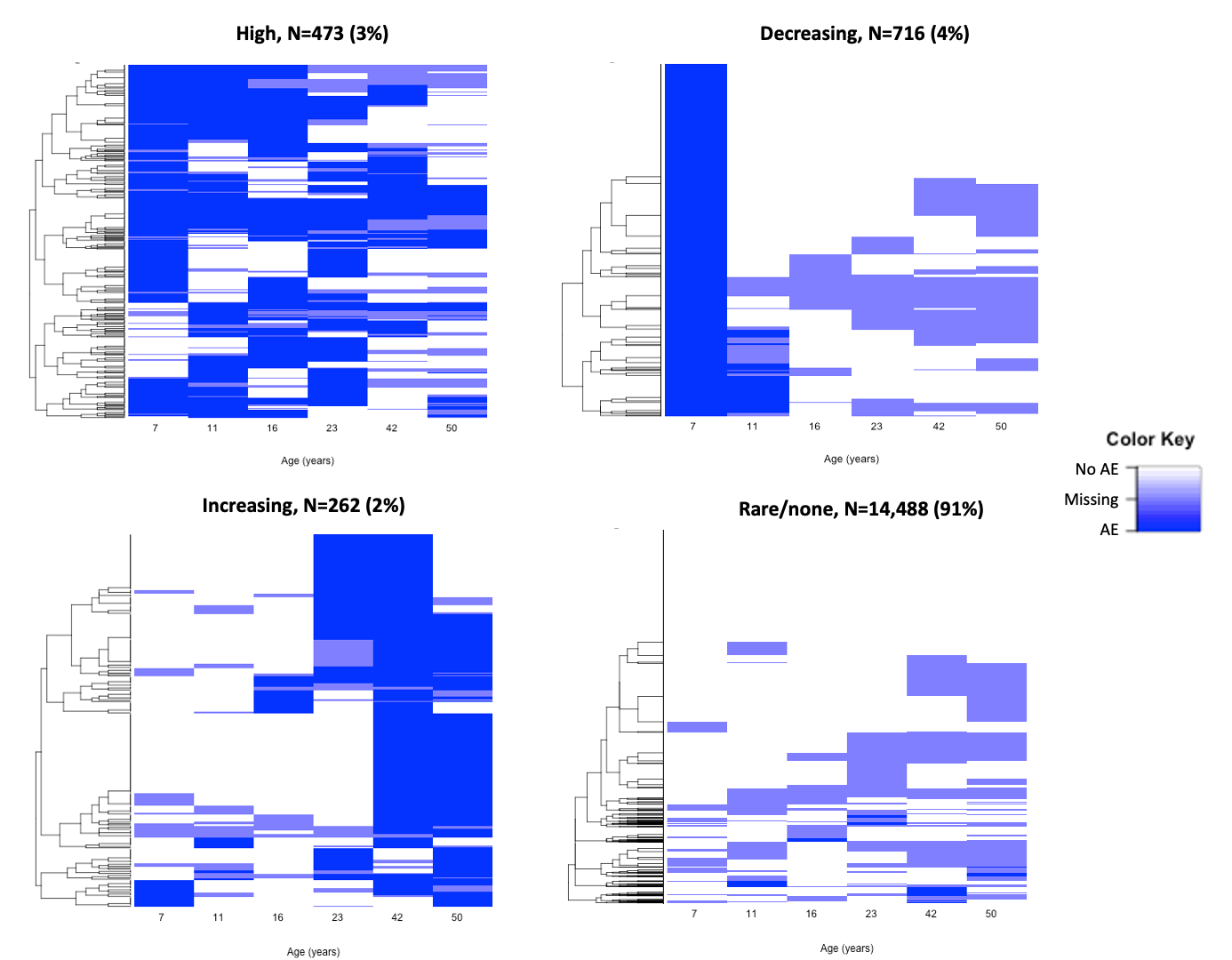
**

**1970 Cohort**

**
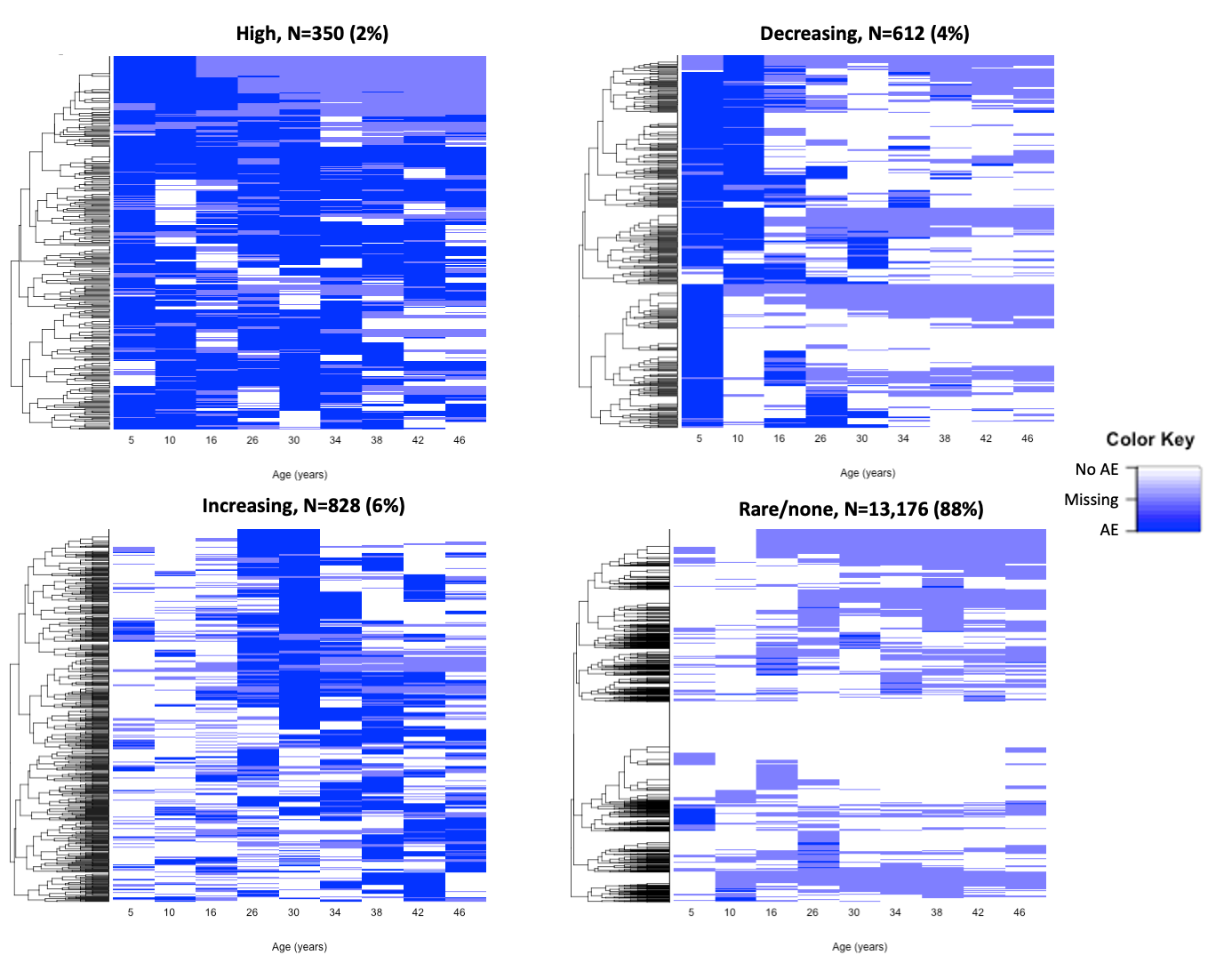
**

**eTable 1. Questions about atopic eczema, asthma, and rhinitis by year and by cohort**

**Eczema questions**

| **1958 Cohort** | | | | | **1970 Cohort** | | | | |
| --- | --- | --- | --- | --- | --- | --- | --- | --- | --- |
| Age | Question | Missing  (N=17,196 at birth) | Positive responses (% of total responses at that wave) | | Age | Question | Missing (N=17,415 at birth) | Positive responses (% of total responses at that wave | |
| Age 7 | Eczema during or prior to past year? | 3,385 | 1,100 | 8% | Age 5 | Eczema during or prior to past year? | 5,013 | 1,495 | 12% |
| Age 11 | Eczema during past year? | 4,432 | 652 | 5% | Age 10 | Eczema during past year? | 4,823 | 887 | 7% |
| Age 16 | Eczema in the past year? | 3,714 | 470 | 3% | Age 16 | Eczema during past year? | 8,296 | 668 | 8% |
| Age 23 | Eczema in the past year? | 5,577 | 634 | 5% | Age 26 | Eczema during past year? | 8,863 | 963 | 12% |
|  |  |  |  |  | Age 30 | Eczema during past year? | 6,802 | 978 | 9% |
|  |  |  |  |  | Age 34 | Eczema since last survey? | 8,242 | 662 | 7% |
|  |  |  |  |  | Age 38 | Eczema currently? | 8,992 | 604 | 7% |
| Age 42 | Eczema in the past year? | 6,627 | 812 | 8% | Age 42 | Eczema since last survey? | 8,103 | 566 | 6% |
|  |  |  |  |  | Age 46 | Eczema since last survey | 9,246 | 410 | 5% |
| Age 50 | Eczema currently? | 8,165 | 442 | 5% |  |  |  |  |  |

^a^Note that shaded questions are most similar across cohorts and included in the sensitivity analyses.

**Asthma questions**

| **1958 Cohort** | | **1970 Cohort** | |
| --- | --- | --- | --- |
| Age | Question | Age | Question |
| Age 7 | Ever had asthma attack? |  |  |
| Age 11 | Ever had asthma or wheezy bronchitis? | Age 10 | Asthma during past year? |
| Age 16 | Ever had asthma or wheezy bronchitis? | Age 16 | Had asthma during past year? |
| Age 23 | Asthma or bronchitis in the past year? | Age 26 | Suffered from asthma during past year? |
|  |  | Age 30 | Had asthma during past year? |
|  |  | Age 34 | Asthma attacks in the last 12 months? |
|  |  | Age 38 | Suffers from asthma or wheezy bronchitis currently? |
| Age 42 | Asthma in the past year? | Age 42 | Asthma or wheezy bronchitis since last survey? |
|  |  | Age 46 | Asthma or wheezy bronchitis since last survey? |
| Age 50 | Suffers from asthma or wheezy bronchitis currently? |  |  |

**Hay fever/rhinitis questions**

| **1958 Cohort** | | **1970 Cohort** | |
| --- | --- | --- | --- |
| Age | Question | Age | Question |
| Age 7 | Ever hay fever or sneezing attacks? | Age 5 | Hay fever and sneezing during or prior to past year? |
| Age 11 | Hay fever/rhinitis during past year? | Age 10 | Hay fever during past year? |
| Age 16 | Hay fever in the past year? | Age 16 | Had hay fever/allergic rhinitis during past year? |
| Age 23 | Hay fever in the past year? | Age 26 | Suffered from hay fever during past year? |
|  |  | Age 30 | Had hay fever/allergic rhinitis during past year? |
|  |  | Age 34 | Hay fever during past year? |
|  |  | Age 38 | Suffers from hay fever/sneeze/runny nose currently? |
| Age 42 | Hay fever/allergic rhinitis in the past year? | Age 42 | Hay fever since last survey? |
|  |  | Age 46 | Hay fever since last survey? |
| Age 50 | Suffers from hay fever currently? |  |  |

**eTable 2. Latent class fit statistics**

**1958 Cohort**

| ***Model fit statistics*** | | | | | | | |  |
| --- | --- | --- | --- | --- | --- | --- | --- | --- |
|  | **2 Class Model** |  | **3 Class Model** |  | **4 Class Model** |  | **5 Class Model** | |
| Log likelihood | -14371.63 |  | -13974.91 |  | -13886.37 |  | -13846.54 | |
| Degrees Freedom | 5 |  | 8 |  | 11 |  | 14 | |
| BIC | 28799.22 |  | 28039.37 |  | 27895.86 |  | 27849.77 | |
| Sample size-adjusted BIC | 28783.33 |  | 28013.94 |  | 27860.90 |  | 27805.28 | |
| Entropy | 0.78 |  | 0.73 |  | 0.89 |  | 0.79 | |
| ***Distribution of individuals among subgroups*** | | | | | | | |  |
|  | **N (%)** |  | **N (%)** |  | **N (%)** |  | **N (%)** | |
| Rare/none | 13,807 (87) |  | 13,879 (87) |  | 14,488 (91) |  | 13,278 (83) | |
| Decreasing | - |  | 510 (3) |  | 716 (4) |  | 725 (5) | |
| Increasing high | - |  | - |  | - |  | 151 (1) | |
| Increasing low | 2,132 (13) |  | 1,550 (10) |  | 262 (2) |  | 1,430 (9) | |
| High | - |  | - |  | 473 (3) |  | 355 (2) | |

**1970 Cohort**

| ***Model fit statistics*** | | | | | | | |  |
| --- | --- | --- | --- | --- | --- | --- | --- | --- |
|  | **2 Class Model** |  | **3 Class Model** |  | **4 Class Model** |  | **5 Class Model** | |
| Log likelihood | -22788.98 |  | -22461.98 |  | -22196.70 |  | -22159.75 | |
| Degrees Freedom | 5 |  | 8 |  | 11 |  | 14 | |
| BIC | 45635.45 |  | 45015.95 |  | 44519.89 |  | 44480.48 | |
| Sample size-adjusted BIC | 45619.56 |  | 44990.52 |  | 44484.92 |  | 44435.99 | |
| Entropy | 0.93 |  | 0.93 |  | 0.92 |  | 0.91 | |
| ***Distribution of individuals among subgroups*** | | | | | | | |  |
|  | **N (%)** |  | **N (%)** |  | **N (%)** |  | **N (%)** | |
| Rare/none | 13,495 (90) |  | 13,426 (90) |  | 13,176 (88) |  | 13,106 (88) | |
| Decreasing | - |  | 614 (4) |  | 612 (4) |  | 545 (4) | |
| Increasing high | - |  | - |  | - |  | 134 (1) | |
| Increasing low | 1,471 (10) |  | 926 (6) |  | 828 (6) |  | 816 (5) | |
| High | - |  | - |  | 350 (2) |  | 365 (2) | |

**1958 Cohort** (using only five comparable data inputs across cohorts)

| ***Model fit statistics*** | | | | | | | |  |
| --- | --- | --- | --- | --- | --- | --- | --- | --- |
|  | **2 Class Model** |  | **3 Class Model** |  | **4 Class Model** |  | **5 Class Model** | |
| Log likelihood | -12784.14 |  | -12387.77 |  | -12330.09 |  | -12324.42 | |
| Degrees Freedom | 5 |  | 8 |  | 11 |  | 13 | |
| BIC | 25623.56 |  | 24863.99 |  | 24781.80 |  | 24792.58 | |
| Sample size-adjusted BIC | 25607.67 |  | 24838.57 |  | 24746.84 |  | 24751.26 | |
| Entropy | 0.77 |  | 0.69 |  | 0.75 |  | 0.79 | |
| ***Distribution of individuals among subgroups*** | | | | | | | |  |
|  | **N (%)** |  | **N (%)** |  | **N (%)** |  | **N (%)** | |
| Rare/none | 13,975 (88) |  | 14,197 (89) |  | 14,049 (88) |  | 14,049 (88) | |
| Decreasing high | - |  | 558 (4) |  | - |  | 188 (1) | |
| Decreasing low | - |  | - |  | 171 (1) |  | 117 (1) | |
| Increasing | 1,951 (12) |  | 1,171 (7) |  | 1,388 (9) |  | 1,403 (9) | |
| High | - |  | - |  | 318 (2) |  | 169 (1) | |

**1970 Cohort** (using only five comparable data inputs across cohorts)

| ***Model fit statistics*** | | | | | | | |  |
| --- | --- | --- | --- | --- | --- | --- | --- | --- |
|  | **2 Class Model** |  | **3 Class Model** |  | **4 Class Model** |  | **5 Class Model** | |
| Log likelihood | -15547.25 |  | -15463.6 |  | -15326.87 |  | -15321.36 | |
| Degrees Freedom | 5 |  | 8 |  | 11 |  | 14 | |
| BIC | 31149.76 |  | 31015.62 |  | 30775.31 |  | 30797.44 | |
| Sample size-adjusted BIC | 31133.87 |  | 30990.19 |  | 30740.35 |  | 30752.96 | |
| Entropy | 0.92 |  | 0.94 |  | 0.94 |  | 0.75 | |
| ***Distribution of individuals among subgroups*** | | | | | | | |  |
|  | **N (%)** |  | **N (%)** |  | **N (%)** |  | **N (%)** | |
| Rare/none | 13,801 (93) |  | 13,752 (92) |  | 13,168 (88) |  | 5,642 (38) | |
| Decreasing | 1,084 (7) |  | 109 (1) |  | 659 (4) |  | 671 (5) | |
| Increasing high | - |  | 1,024 (7) |  | 109 (1) |  | 111 (1) | |
| Increasing low | - |  | - |  | - |  | 7,612 (51) | |
| High | - |  | - |  | 949 (6) |  | 849 (6) | |

Notes: Lower log likelihood and BIC indicate better model fit. Higher entropy score indicates better class discrimination

## **eTable 3: Average classification probabilities by most likely disease trajectory subtype for the four-class solution**

1958 Cohort

|  | Subtype | | | |
| --- | --- | --- | --- | --- |
| Most likely subtype (assigned class) | Prob  (High) | Prob  (Decreasing) | Prob  (Increasing) | Prob  (Rare/No disease) |
| High | **0.832** | 0.031 | 0.076 | 0.062 |
| Decreasing | 0.075 | **0.658** | 0.018 | 0.250 |
| Increasing | 0.090 | 0.000 | **0.791** | 0.119 |
| Rare/No disease | 0.004 | 0.019 | 0.027 | **0.950** |

1970 Cohort

|  | Subtype | | | |
| --- | --- | --- | --- | --- |
| Most likely subtype (assigned class) | Prob  (High) | Prob  (Decreasing) | Prob  (Increasing) | Prob  (Rare/No disease) |
| High | **0.804** | 0.102 | 0.093 | 0.001 |
| Decreasing | 0.078 | **0.753** | 0.071 | 0.097 |
| Increasing | 0.050 | 0.052 | **0.791** | 0.107 |
| Rare/No disease | 0.001 | 0.021 | 0.035 | **0.943** |

**Notes**: Average probabilities of subtype membership, according to their most likely subtype (rows). Numbers close to 1 indicate high confidence in the classification.

**eTable 4. Association between early life factors and atopic eczema activity subtypes by cohort**

Panel A: 1958 Cohort (Total N across all classes = 15,939)

|  | High vs  Rare/no AE | Decreasing vs  Rare/no AE | Increasing vs  Rare/no AE | High vs  Decreasing AE | Increasing vs Decreasing AE |
| --- | --- | --- | --- | --- | --- |
|  | Odds Ratio (95% CI) | | | | |
| Sex |  |  |  |  |  |
| Male | Reference | Reference | Reference | Reference | Reference |
| Female | **1.63 (1.35, 1.97)** | **0.81 (0.70, 0.95)** | **1.73 (1.34, 2.22)** | **2.01 (1.58, 2.55)** | **2.12 (1.58, 2.84)** |
| Ethnicity |  |  |  |  |  |
| European, Caucasian | Reference | Reference | Reference | Reference | Reference |
| Other | 0.56 (0.17, 1.82) | 0.83 (0.39, 1.76) | 1.43 (0.53, 3.85) | 0.67 (0.17, 2.71) | 1.72 (0.50, 5.94) |
| Region of early childhood residence |  |  |  |  |  |
| Southern England | Reference | Reference | Reference | Reference | Reference |
| Central England/Wales | 1.11 (0.89, 1.39) | 0.89 (0.74, 1.07) | 1.06 (0.78, 1.44) | 1.24 (0.93, 1.66) | 1.19 (0.83, 1.69) |
| N. England/Scotland | 0.78 (0.62, 0.98) | **0.78 (0.65, 0.93)** | **0.86 (0.64, 1.16)** | 1.00 (0.75, 1.33) | 1.10 (0.78, 1.56) |
| Highest social class in childhood^a^ |  |  |  |  |  |
| I/II | Reference | Reference | Reference | Reference | Reference |
| III | **0.74 (0.61, 0.91)** | **0.82 (0.69, 0.97)** | 0.86 (0.66, 1.14) | 0.90 (0.70, 1.17) | 1.06 (0.77, 1.45) |
| IV/V | **0.59 (0.41, 0.85)** | **0.53 (0.38, 0.73)** | 0.72 (0.45, 1.16) | 1.12 (0.69, 1.81) | 1.37 (0.78, 2.41) |
| Household size in early childhood |  |  |  |  |  |
| <=3 persons | Reference | Reference | Reference | Reference | Reference |
| 4+ persons | 1.15 (0.80, 1.65) | 0.85 (0.66, 1.10) | 0.92 (0.59, 1.42) | 1.35 (0.87, 2.08) | 1.07 (0.65, 1.77) |
| In utero smoke exposure |  |  |  |  |  |
| No | Reference | Reference | Reference | Reference | Reference |
| Any | 0.89 (0.71, 1.12) | 1.03 (0.86, 1.24) | 1.01 (0.76, 1.35) | 0.86 (0.65, 1.14) | 0.98 (0.70, 1.37) |
| Childhood smoke exposure |  |  |  |  |  |
| No | Reference | Reference | Reference | Reference | Reference |
| Any | 0.90 (0.71, 1.13) | 0.91 (0.73, 1.14) | 0.90 (0.66, 1.22) | 0.98 (0.72, 1.33) | 0.98 (0.68, 1.42) |
| Birth weight |  |  |  |  |  |
| Per kg increase | 1.09 (0.91, 1.32) | 1.04 (0.90, 1.21) | 1.06 (0.83, 1.36) | 1.05 (0.83, 1.32) | 1.02 (0.76, 1.36) |
| Breastfeeding |  |  |  |  |  |
| No | Reference | Reference | Reference | Reference | Reference |
| Any | **1.38 (1.10, 1.73)** | **1.27 (1.06, 1.51)** | **1.42 (1.05, 1.93)** | 1.09 (0.83, 1.44) | 1.12 (0.80, 1.59) |

^a^Registrar General’s social class: I Professional, II Managerial and technical; III Skilled; IV Partly-skilled; V Unskilled.

Panel B: 1970 Cohort (Total N across all classes = 14,966)

|  | High vs  Rare/no AE | Decreasing vs  Rare/no AE | Increasing vs  Rare/no AE | High vs  Decreasing AE | Increasing vs Decreasing AE |
| --- | --- | --- | --- | --- | --- |
|  | Odds Ratio (95% CI) | | | | |
| Sex |  |  |  |  |  |
| Male | Reference | Reference | Reference | Reference | Reference |
| Female | **1.97 (1.58, 2.46)** | 0.99 (0.84, 1.17) | **1.80 (1.56, 2.08)** | **1.99 (1.52, 2.61)** | **1.82 (1.47, 2.25)** |
| Ethnicity |  |  |  |  |  |
| European, Caucasian | Reference | Reference | Reference | Reference | Reference |
| Other | 1.12 (0.63, 1.99) | 1.16 (0.78, 1.74) | 0.82 (0.55, 1.22) | 0.97 (0.49, 1.89) | 0.70 (0.41, 1.21) |
| Region of early childhood residence |  |  |  |  |  |
| Southern England | Reference | Reference | Reference | Reference | Reference |
| Central England/Wales | 1.03 (0.77, 1.38) | 0.83 (0.68, 1.02) | 0.90 (0.74, 1.08) | 1.24 (0.88, 1.75) | 1.08 (0.82, 1.41) |
| N. England/Scotland | 0.94 (0.71, 1.23) | **0.65 (0.52, 0.79)** | **0.83 (0.69, 0.99)** | 1.45 (1.04, 2.02) | 1.28 (0.98, 1.67) |
| Highest social class in childhood^a^ |  |  |  |  |  |
| I/II | Reference | Reference | Reference | Reference | Reference |
| III | **0.67 (0.54, 0.84)** | **0.83 (0.70, 0.99)** | 0.94 (0.81, 1.10) | 0.81 (0.61, 1.07) | 1.13 (0.90, 1.42) |
| IV/V | **0.41 (0.23, 0.71)** | **0.66 (0.46, 0.94)** | 0.97 (0.73, 1.28) | 0.62 (0.32, 1.20) | 1.47 (0.94, 2.30) |
| Household size in early childhood |  |  |  |  |  |
| <=3 persons | Reference | Reference | Reference | Reference | Reference |
| 4+ persons | 0.89 (0.62, 1.28) | 1.05 (0.79, 1.40) | 0.93 (0.73, 1.20) | 0.85 (0.54, 1.33) | 0.89 (0.62, 1.27) |
| In utero smoke exposure |  |  |  |  |  |
| No | Reference | Reference | Reference | Reference | Reference |
| Any | 0.78 (0.61, 1.01) | 0.99 (0.82, 1.19) | 0.88 (0.75, 1.04) | 0.79 (0.58, 1.08) | 0.89 (0.70, 1.14) |
| Childhood smoke exposure |  |  |  |  |  |
| No | Reference | Reference | Reference | Reference | Reference |
| Any | 1.03 (0.79, 1.34) | 1.03 (0.84, 1.25) | 0.94 (0.79, 1.11) | 1.00 (0.73, 1.39) | 0.91 (0.71, 1.18) |
| Parent atopy |  |  |  |  |  |
| No | Reference | Reference | Reference | Reference | Reference |
| Any | **2.54 (2.00, 3.23)** | **2.52 (2.11, 3.01)** | **1.38 (1.16, 1.65)** | 1.01 (0.75, 1.35) | **0.55 (0.43, 0.70)** |
| Birth weight |  |  |  |  |  |
| Per kg increase | 1.06 (0.85, 1.31) | 0.95 (0.81, 1.11) | 0.89 (0.77, 1.02) | 1.11 (0.86, 1.44) | 0.93 (0.76, 1.15) |
| Breastfeeding |  |  |  |  |  |
| No | Reference | Reference | Reference | Reference | Reference |
| Any | **1.36 (1.09, 1.71)** | 1.12 (0.94, 1.33) | 1.12 (0.96, 1.30) | 1.21 (0.92, 1.60) | 1.00 (0.80, 1.25) |

^a^Registrar General’s social class: I Professional, II Managerial and technical; III Skilled; IV Partly-skilled; V Unskilled.

**eTable 5: Proportions of individuals with atopic eczema, asthma, and rhinitis within each disease trajectory subtype with by cohort**

­­

| **1958 Cohort** |  | Atopic eczema subclass | | | |
| --- | --- | --- | --- | --- | --- |
|  | Overall | High | Decreasing | Increasing | Rare/None |
| Age 7  Eczema  Asthma  Rhinitis | 8%  3%  6% | 80%  15%  18% | 100%  13%  12% | 8%  3%  7% | 0.1%  2%  5% |
| Age 11  Eczema  Asthma  Rhinitis | 5%  12%  8% | 69%  33%  27% | 19%  24%  17% | 5%  12%  9% | 2%  11%  7% |
| Age 16  Eczema  Asthma  Rhinitis | 3%  12%  12% | 69%  32%  34% | 0%  21%  22% | 10%  11%  15% | 1%  11%  11% |
| Age 23  Eczema  Asthma  Rhinitis | 5%  4%  17% | 74%  14%  39% | 0%  8%  23% | 48%  8%  30% | 2%  4%  15% |
| Age 42  Eczema  Asthma  Rhinitis | 8%  6%  16% | 46%  17%  32% | 0%  9%  19% | 92%  14%  27% | 4%  5%  15% |
| Age 50  Eczema  Asthma  Rhinitis | 5%  10%  14% | 29%  24%  27% | 0%  13%  15% | 74%  20%  26% | 2%  9%  13% |

| **1970 Cohort** |  | Atopic eczema subclass | | | |
| --- | --- | --- | --- | --- | --- |
|  | Overall | High | Decreasing | Increasing | Rare/None |
| Age 5  Eczema  Asthma  Rhinitis | 12%  -  4% | 93%  -  15% | 96%  -  14% | 10%  -  6% | 6%  -  4% |
| Age 10  Eczema  Asthma  Rhinitis | 7%  3%  11% | 83%  14%  34% | 59%  12%  25% | 11%  4%  16% | 2%  2%  10% |
| Age 16  Eczema  Asthma  Rhinitis | 8%  9%  25% | 84%  34%  46% | 38%  16%  42% | 18%  14%  32% | 3%  7%  23% |
| Age 26  Eczema  Asthma  Rhinitis | 12%  8%  21% | 86%  24%  40% | 29%  16%  29% | 58%  15%  29% | 4%  6%  19% |
| Age 30  Eczema  Asthma  Rhinitis | 9%  7%  20% | 87%  21%  39% | 15%  14%  27% | 65%  13%  29% | 2%  6%  18% |
| Age 34  Eczema  Asthma  Rhinitis | 7%  4%  22% | 83%  8%  40% | 9%  6%  30% | 52%  8%  32% | 1%  3%  20% |
| Age 38  Eczema  Asthma  Rhinitis | 7%  10%  20% | 77%  29%  41% | 4%  16%  28% | 55%  20%  30% | 1%  9%  18% |
| Age 42  Eczema  Asthma  Rhinitis | 6%  9%  20% | 68%  24%  42% | 2%  11%  28% | 49%  15%  28% | 1%  8%  18% |
| Age 46  Eczema  Asthma  Rhinitis | 5%  12%  20% | 61%  30%  42% | 1%  17%  27% | 37%  18%  29% | 1%  10%  19% |

**eTable 6. Complete case analysis: Association between early life factors and atopic eczema activity subtypes from for 1958 and 1970 meta-analysis (Total N across all classes = 19,888)**

|  | High vs  Rare/no AE | Decreasing vs  Rare/no AE | Increasing vs  Rare/no AE | High vs  Decreasing AE | Increasing vs Decreasing AE |
| --- | --- | --- | --- | --- | --- |
|  | Odds Ratio (95% CI) | | | | |
| Sex |  |  |  |  |  |
| Male | Reference | Reference | Reference | Reference | Reference |
| Female | **1.76 (1.48, 2.10)** | 0.96 (0.84, 1.09) | **1.82 (1.58, 2.11)** | **1.84 (1.49, 2.29)** | **1.91 (1.57, 2.31)** |
| Ethnicity |  |  |  |  |  |
| European, Caucasian | Reference | Reference | Reference | Reference | Reference |
| Other | 0.83 (0.47, 1.46) | 1.02 (0.68, 1.52) | 0.64 (0.41, 1.01) | 0.81 (0.41, 1.61) | 0.63 (0.35, 1.14) |
| Region of early childhood residence |  |  |  |  |  |
| Southern England | Reference | Reference | Reference | Reference | Reference |
| Central England/Wales | 1.05 (0.86, 1.30) | **0.83 (0.71, 0.98)** | 0.90 (0.76, 1.08) | 1.27 (0.98, 1.64) | 1.08 (0.86, 1.37) |
| N. England/Scotland | **0.78 (0.63, 0.96)** | **0.63 (0.54, 0.74)** | **0.78 (0.66, 0.92)** | 1.24 (0.96, 1.61) | 1.24 (0.99, 1.56) |
| Highest social class in childhood^a^ |  |  |  |  |  |
| I/II | Reference | Reference | Reference | Reference | Reference |
| III | **0.76 (0.64, 0.91)** | **0.79 (0.69, 0.91)** | 0.90 (0.78, 1.05) | 0.97 (0.77, 1.21) | 1.15 (0.94, 1.40) |
| IV/V | **0.52 (0.35, 0.78)** | **0.65 (0.48, 0.87)** | 0.85 (0.63, 1.14) | 0.80 (0.49, 1.32) | 1.30 (0.86, 1.96) |
| Household size in early childhood |  |  |  |  |  |
| <=3 persons | Reference | Reference | Reference | Reference | Reference |
| 4+ persons | 0.94 (0.70, 1.25) | 1.00 (0.79, 1.26) | 0.96 (0.76, 1.21) | 0.94 (0.65, 1.35) | 0.96 (0.70, 1.33) |
| In utero smoke exposure |  |  |  |  |  |
| No | Reference | Reference | Reference | Reference | Reference |
| Any | **0.76 (0.62, 0.93)** | 0.98 (0.84, 1.14) | 0.92 (0.78, 1.08) | **0.77 (0.60, 0.99)** | 0.93 (0.75, 1.16) |
| Childhood smoke exposure |  |  |  |  |  |
| No | Reference | Reference | Reference | Reference | Reference |
| Any | 0.99 (0.82, 1.20) | 0.98 (84, 1.14) | 0.95 (0.81, 1.12) | 1.01 (0.79, 1.28) | 0.97 (0.78, 1.20) |
| Birth weight |  |  |  |  |  |
| Per kg increase | 1.02 (0.86, 1.20) | 0.95 (0.83, 1.08) | 0.94 (0.82, 1.08) | 1.07 (0.87, 1.32) | 1.00 (0.83, 1.20) |
| Breastfeeding |  |  |  |  |  |
| No | Reference | Reference | Reference | Reference | Reference |
| Any | **1.37 (1.14, 1.65)** | 1.13 (0.98, 1.31) | 1.14 (0.98, 1.32) | 1.21 (0.96, 1.53) | 1.01 (0.82, 1.23) |
| Cohort |  |  |  |  |  |
| 1970 | Reference | Reference | Reference | Reference | Reference |
| 1958 | **1.29 (1.08, 1.55)** | 1.13 (0.97, 1.30) | **0.33 (0.27, 0.39)** | 1.15 (0.91, 1.44) | **0.29 (0.23, 0.37)** |

^a^Registrar General’s social class: I Professional, II Managerial and technical; III Skilled; IV Partly-skilled; V Unskilled
